# Deep sub-angstrom resolution imaging by electron ptychography with misorientation correction

**DOI:** 10.1126/sciadv.abn2275

**Published:** 2022-05-13

**Authors:** Haozhi Sha, Jizhe Cui, Rong Yu

**Affiliations:** 1 National Center for Electron Microscopy in Beijing, Tsinghua University, Beijing 100084, China.; 2 Key Laboratory of Advanced Materials of Ministry of Education of China, Tsinghua University, Beijing 100084, China.; 3 State Key Laboratory of New Ceramics and Fine Processing, Tsinghua University, Beijing 100084, China.; 4 School of Materials Science and Engineering, Tsinghua University, Beijing 100084, China.

## Abstract

Superresolution imaging of solids is essential to explore local symmetry breaking and derived material properties. Electron ptychography is one of the most promising schemes to realize superresolution imaging beyond aberration correction. However, to reach both deep sub-angstrom resolution imaging and accurate measurement of atomic structures, it is still required for the electron beam to be nearly parallel to the zone axis of crystals. Here, we report an efficient and robust method to correct the specimen misorientation in electron ptychography, giving deep sub-angstrom resolution for specimens with large misorientations. The method largely reduces the experimental difficulties of electron ptychography and paves the way for widespread applications of ptychographic deep sub-angstrom resolution imaging.

## Teaser

Adaptive propagator ptychography enables local structure analysis with deep-subangstrom resolution and subpicometer accuracy.

## INTRODUCTION

As an important degree of freedom in solids, atomic lattice structure contains numerous information that is directly correlated with physical and chemical properties of functional materials (*1*–*3*). For example, relative displacements between cations and anions determine the electric polarization of polar crystals. Local lattice distortions play a key role in entropy-stabilized structural and functional materials (*4*, *5*). Surface relaxations and reconstructions greatly affect the catalytic performances of nanocrystals (*6*, *7*). To obtain the structure information with high spatial resolution, atomically resolved electron microscopy is usually needed. However, a ubiquitous problem in quantitative microscopic analysis is how to get reliable and accurate results when the geometrical changes are small (e.g., less than 10 pm) (*8*, *9*). A nearly inevitable factor that hinders deep sub-angstrom resolution and high accuracy in lattice structure measurements is the misorientation between electron beam and zone axis of crystals, including extrinsic misorientation, like specimen mistilt, and intrinsic misorientations, like those close to interfaces and dislocations. Misorientation has substantial influence on nearly all the imaging modes, including high-resolution transmission electron microscopy (HRTEM) (*10*, *11*), scanning transmission electron microscopy (STEM) with high-angle annular dark field (HAADF) (*12*–*15*), annular bright field (ABF) (*8*, *9*, *16*, *17*), bright field (*16*), and differential phase contrast (DPC) (*18*, *19*). It can not only introduce artificial contrast and relative shifts between different types of atoms but also reduce atomic column visibility, obscuring important structural changes such as ferroelectric shift and local lattice distortion. For example, about 11.9 pm of artificial displacement between O and Sr/TiO columns would be induced in 25-nm-thick SrTiO3 under a mistilt of 6 mrad (*8*). Oxygen octahedral tilt deviates about 3° from its real value in BiFeO_3_ of 16 nm under a mistilt of 5 mrad (*16*). However, structural variations in many materials are as tiny as several picometers, such as some high-entropy alloys (*20*) and improper ferroelectrics (*21*). In this way, mapping the lattice structure with high accuracy in the presence of misorientation is of great practical value.

Electron ptychography has received broad interest as a superresolution imaging technique (*22*, *23*). In electron microscopy, structure information is usually encoded in the phase of electron waves and lost in the signal collecting process. On the basis of a set of two-dimensional (2D) coherent diffraction patterns acquired at each scan position (*24*, *25*) (so-called 4D dataset) and proper algorithms (*26*–*31*), ptychography can recover the lost phases (*32*, *33*). In the early stage of electron ptychography, phase recovery is mainly applied to thin materials, which scatter electrons weakly and thus can be modeled by a single slice (*34*–*38*). Relying on large data redundancy, algorithms can recover the beam function simultaneously (*29*), pushing the lateral resolution to 0.39 Å in 2D materials (*34*). In addition, the good contrast linearity enables ptychography to show both heavy and light atoms with high interpretability (*37*, *39*, *40*). Separating electron beams to overlapping illuminated areas and using every pixel with reasonable counts to do reconstruction, ptychography can get higher signal-to-noise ratio and higher spatial resolution than DPC at the same level of dose (*35*, *41*–*43*).

Recently, by using multislice electron ptychography, Chen *et al.* (*37*) overcame the multiple scattering effects and realized deep sub-angstrom resolution in thick samples. Dividing a thick sample into thin slices and describing the electron propagation between adjacent slices by the Fresnel propagator, multislice ptychography can recover 3D phase distributions with some depth resolution. On the basis of the 3D imaging ability, a misorientation correction scheme was also suggested. For samples tilted away from the zone axis, standard multislice ptychography is expected to obtain a series of phase images with depth-dependent horizontal relative shifts. After finishing the reconstruction, the phase images can be aligned via cross-correlation. The misorientation effect can be reduced. However, detailed analysis shown below indicates that the post-alignment scheme does not always live up to the expectation, because the misorientation effect is present in each single slice. This leaves misorientation correction a challenge in the way to deep sub-angstrom resolution imaging and quantitative structure analysis.

In standard multislice ptychography, the Fresnel propagator is fixed throughout the reconstruction process. Here, making the Fresnel propagator adaptive in ptychographic reconstruction, we have realized reliable and efficient elimination of misorientations without any extra postprocessing. Tested with simulation and experimental datasets, the proposed method is demonstrated to be capable of correcting specimen mistilt even with relatively thick slices when the standard multislice ptychography fails. In addition, our method is less sensitive to detector noise and overlap ratio than standard multislice ptychography. The method effectively alleviates the experimental challenge and improves the spatial resolution and accuracy of quantitative structure analysis. The positions of atomic columns can be measured with subpicometer accuracy even with large misorientations.

## RESULTS

### Theory of adaptive propagator ptychography

Treated as an optimization problem, ptychographic reconstruction can be solved by various well-developed gradient-based methods. During optimization, phases are recovered by minimizing the loss function that describes the difference between the resultant diffraction intensities and the measured ones. This loss function can be formulated as follows (*30*, *31*, *44*)

$$ℒ=\sum_{j}{\left\||ℱ\{\varphi_{\text{ext},j}\}|-\sqrt{I_{j}}\right\|}_{F}^{2}$$ (1)

where *j* is the index of scanning positions. ∣∙∣ calculates element-wise modulus of a matrix, ℱ{∙} denotes the Fourier transformation, and ‖∙‖*_F_* gives the Frobenius norm. φ_ext_ is the exit wave to be optimized, and *I* is the experimentally measured diffraction intensity. When the object is too thick for the phase object approximation, it should be divided into multiple slices. The propagation of electron wave between adjacent slices is described by the Fresnel propagator

$$p(k)=\text{exp}(-i\pi ∆z\lambda {|k|}^{2})$$ (2)

where Δ*z* is the slice thickness, λ is the electron wavelength, and ***k*** is the 2D spatial frequency with *x* and *y* components (*k_x_*, *k_y_*). In standard ptychography, the propagator remains fixed during the reconstruction process.

It is noted that the propagator of Eq. 2 can be modified to include the sample tilt in the multislice simulation of dynamic diffraction (*45*–*47*)

$$p(k;∆z,\theta )=\text{exp}[-i\pi ∆z(\lambda {|k|}^{2}-2k_{x}\text{tan}\;\theta_{x}-2k_{y}\text{tan}\;\theta_{y})]$$ (3)

where **θ** = (θ*_x_*, θ*_y_*) is the tilt angle. In this work, the propagator of Eq. 3 is adopted and made adaptive during the ptychographic reconstruction process, with the tilt angle **θ** and slice thickness Δ*z* being optimized. The method is called adaptive propagator ptychography (APP). Detailed formulas are given in the Supplementary Materials (*48*). For comparison, the standard multislice ptychography using fixed propagator is called fixed propagator ptychography (FPP).

**Fig. 1.**
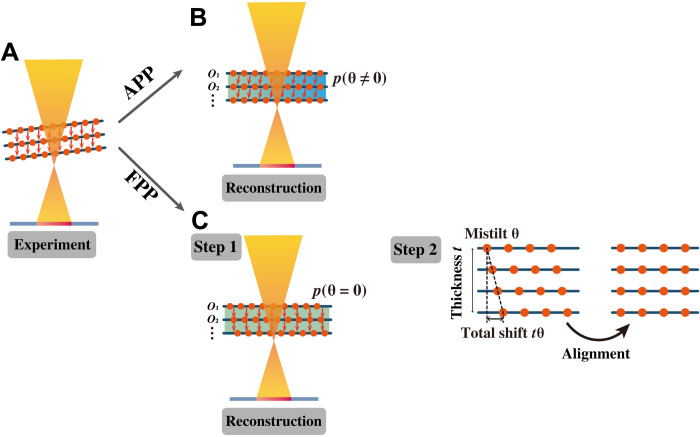
Schematics of multislice ptychography with adaptive propagator (APP) and fixed propagator (FPP). (**A**) Experiment with sample tilt. The orange arrows indicate the propagation direction of electron wave inside the sample. (**B**) APP reconstruction, with the tilt angle θ being optimized. (**C**) FPP reconstruction plus post-alignment.

The two misorientation-correction methods are compared in Fig. 1. For the APP method, the sample tilt in an experiment is included in the electron propagation function, which is then optimized during ptychographic reconstruction (Fig. 1B). For the FPP method, however, the sample tilt is not included in the reconstruction stage but corrected via an aligning process after the reconstruction (Fig. 1C). We will show that the APP method is an efficient way to eliminate misorientation, while FPP can only partly reduce misorientation, especially under thick slices and large misorientation.

### Simulation demonstration of APP

**Fig. 2.**
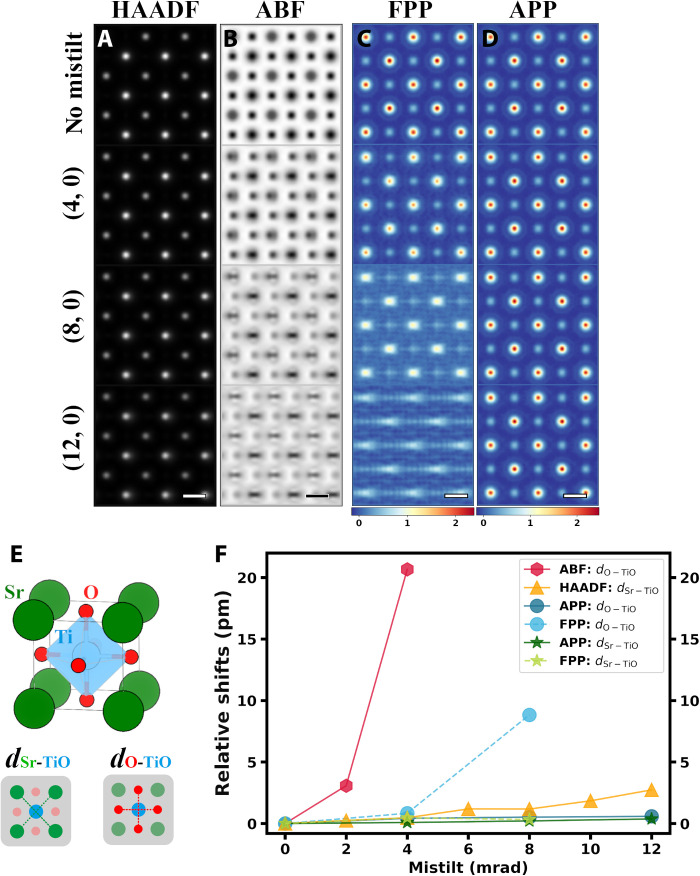
Comparison of conventional STEM imaging modes and ptychography under different magnitudes of misorientation. (**A**) HAADF images, (**B**) ABF images, (**C**) FPP phase images, and (**D**) APP phase images. Scale bars, 2 Å. For APP and FPP, only the middle slice is shown. The mistilt lies in the *x* direction, being 0, 4, 8, and 12 mrad from top to bottom. (**E**) Structure of cubic SrTiO_3_ and illustrations of *d*_Sr-TiO_ and *d*_O-TiO_. (**F**) Relative shifts of atomic columns obtained from HAADF, ABF, FPP, and APP images.

To give a first impression of how misorientation influences structure measurement and to compare the APP and FPP methods, we conduct simulations for cubic SrTiO_3_. Cubic SrTiO_3_ shows no ferroelectric displacements, so the accuracy of displacement measurements can be calculated definitely. Conventional STEM imaging (HAADF and ABF) and 4D datasets are simulated under a series of mistilt angles. Two quantities are measured from STEM images and recovered phases, i.e., the relative shift *d*_Sr-TiO_ (or *d*_O-TiO_) of the TiO column from the center of the four nearest Sr (or O) neighbors. For both APP and FPP, the sample is cut into five slices, and the slice thickness is about 2 nm. Details of the simulations, reconstruction parameters, and effect of noise and probe aberrations are given in the Supplementary Materials. Noise is not included in the data shown in Fig. 2.

Same with previous research, positions of the lighter atom columns are more sensible to misorientation than the heavier ones in ABF images, leading to obvious artifacts in image contrast and relative displacements. Although more robust to misorientation compared to ABF, positional accuracy of HAADF images still deteriorates as the mistilt angle increases. Under a mistilt of 12 mrad, HAADF images have about 2.5 pm of deviation in polarization even when no noise is present (Fig. 2F). Phases of the middle slices recovered from FPP show obviously stretched atomic columns, while APP generates high-quality phase images under all the tested mistilt angles. Even with a misfit of 12 mrad, phases recovered via APP give accurate *d*_Sr-TiO_ and *d*_O-TiO_ with an error smaller than 1 pm. In addition, APP overperforms FPP in the presence of large detector noise and small overlap ratio (figs. S1 and S2).

### Experimental application of APP

**Fig. 3.**
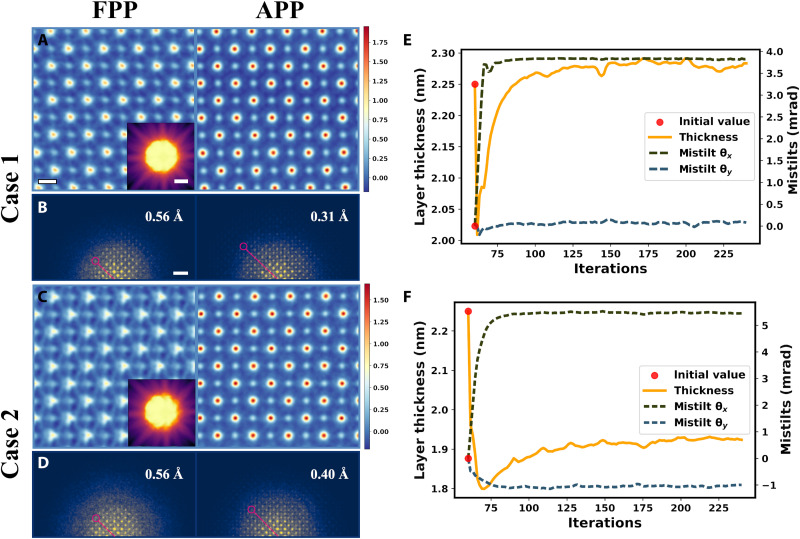
Reconstruction via FPP and APP on experimental datasets with misorientation. (**A** and **C**) Phase images averaged over slices recovered with FPP after post-alignment (left) and APP (right). Scale bar, 2 Å. The insets are PACBEDs. (**B** and **D**) Diffractograms corresponding to (A) and (C). Scale bar, 1 Å^−1^. (**E** and **F**) APP optimization processes of mistilt and thickness corresponding to case 1 and case 2, respectively.

The APP method is applied to experimental datasets of SrTiO_3_ under different magnitudes of misorientation, which are labeled with cases 1 and 2 in Fig. 3. In both cases, samples are cut into 10 slices during reconstruction.

The Kikuchi band in the position-averaged convergent beam electron diffraction (PACBED) in Fig. 3A indicated an obvious tilt of the sample from the <001> zone axis. The phases averaged over the recovered slices are displayed in Fig. 3 (A and C). Using FPP, the recovered phases are of low quality, even after post-alignment. The O atomic columns are more vulnerable to misorientation, leading to their severe stretch in the mistilt direction. The information limit in the diffractogram (Fig. 3B) of the phase is anisotropic. In the direction of the red line, the information limit is about 1.8 Å^−1^, corresponding to 0.56 Å in real space. For larger misorientation (case 2 shown in Fig. 3, C and D), both light and heavy atomic columns distort and the information transfer is damped more strongly in the high-frequency region. Such influence is detrimental to the quantitative characterization of atomic structures.

Using APP, the influence of misorientation can be effectively eliminated. For case 1, the thickness and mistilt (θ*_x_,* θ*_y_*) are determined to be 23 nm and (3.8, 0.1) mrad, respectively (Fig. 3E). For case 2, the thickness and mistilt are 19 nm and (5.4, −1) mrad, respectively (Fig. 3F). The robustness of thickness determination with different initial values is tested, and the results are given in the Supplementary Materials. As shown in Fig. 3 (A and C), the qualities of the phase images recovered with APP are obviously improved over FPP. The information limit becomes more isotropic (Fig. 3, B and D). It is now 3.24 Å^−1^, corresponding to 0.31 Å in real space.

**Fig. 4.**
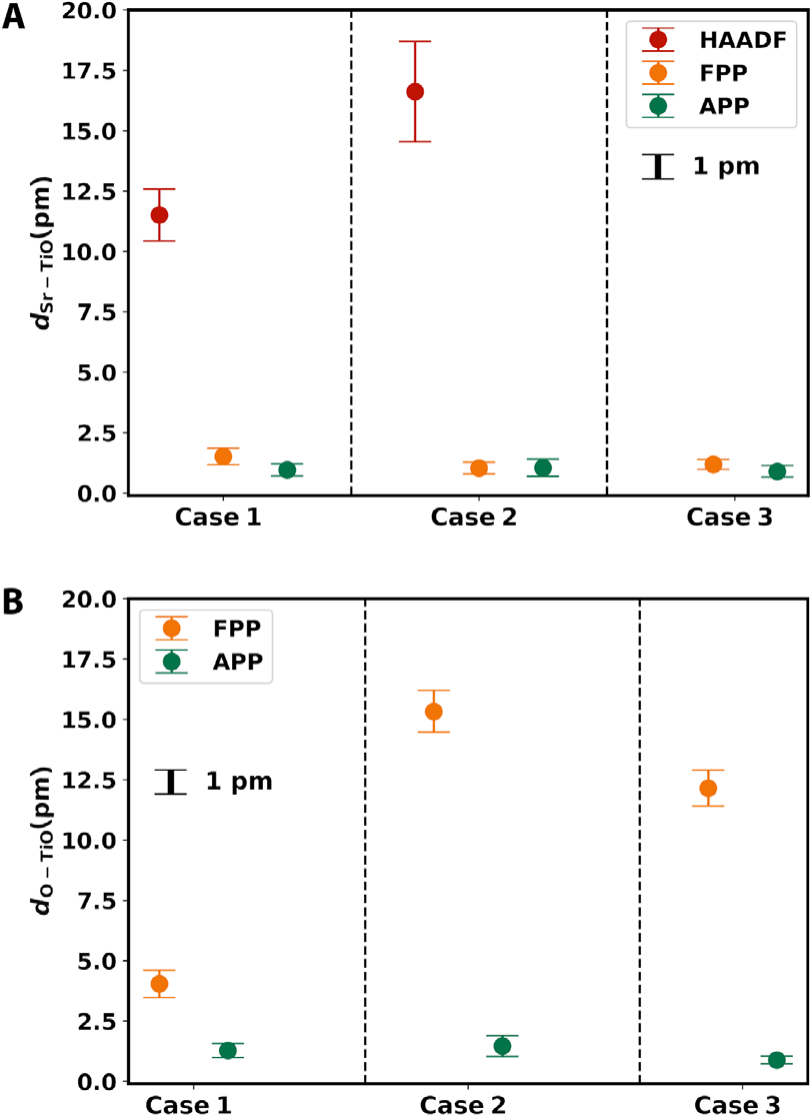
Relative shifts between atomic columns. (**A**) *d*_Sr-TiO_ and (**B**) *d*_O-TiO_ measured from recovered phases of FPP and APP. For *d*_Sr-TiO_ between heavy atomic columns, the values measured from the STEM-HAADF images are also shown for comparison.

To investigate the benefit of misorientation correction on atomic structure analysis, we measured *d*_Sr-TiO_ and *d*_O-TiO_ from the recovered phases. *d*_Sr-TiO_ was also calculated from the HAADF images taken under the same conditions (fig. S3). As shown in Fig. 4, both FPP and APP achieve much better accuracy and precision than HAADF. Using APP, both quantities have accuracy and precision within 1 pm. The improvement of APP over FPP is much more remarkable for *d*_O-TiO_ than for *d*_Sr-TiO_.

### Slice thickness dependence of misorientation correction

**Fig. 5.**
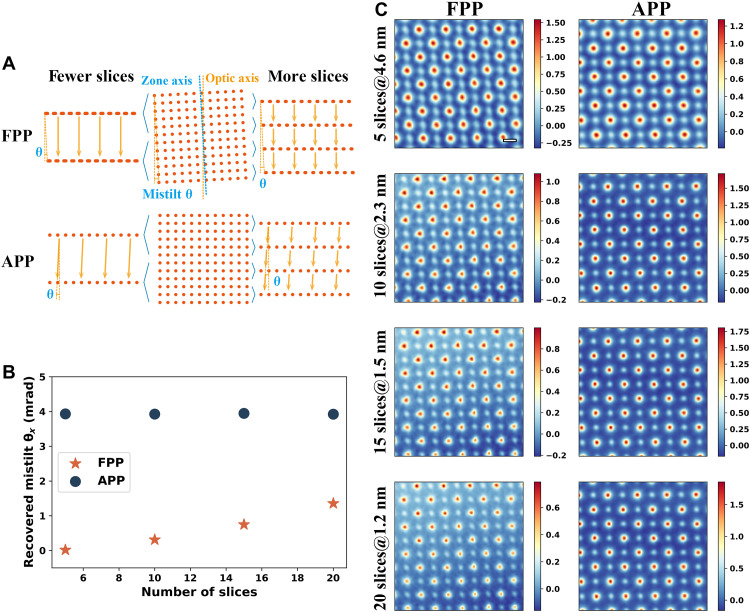
Slice thickness dependence of misorientation correction for FPP and APP. (**A**) Illustration of the effect of using different slice number for FPP and APP. (**B**) Recovered mistilt angle versus slice number. (**C**) Phases averaged over all the slices recovered via FPP and APP. Labels are in the form of “number of slices @ slice thickness.” The dataset of case 1 is used.

The misorientation correction of FPP and APP shows different slice thickness difference. When the zone axis of a sample is tilted away from the optic axis, there are relative horizontal shifts between atomic layers, as shown in Fig. 5A. For FPP, the slices are projected along the optic axis. Because of the limited depth resolution, the slice thickness of multislice electron ptychography is usually 1 to 2 nm. A slice would include several atomic layers; their overlap along the optic axis leads to the stretch of atomic columns. The misorientation-induced stretch can only be eliminated for slices as thin as a single atomic layer, which has not been reached. Therefore, the slice thickness dependence of mistilt is observed for FPP. As shown in Fig. 5C, the recovered mistilt is smaller for thicker slices.

For APP, the slices are projected along the zone axis and the misorientation is included in the propagator. Therefore, the mistilt angle is independent of slice thickness. As shown in Fig. 5B, the tilt angle remains almost the same at different slice thickness or number of slices. More discussions comparing FPP and APP are given in the Supplementary Materials.

### Extension of APP and potential applications

If different oriented samples exist in the field of view, APP shows potential to be extended to simultaneously image the zone axis structure of the whole region. The tilt angle in the propagator for each scan position can be optimized separately to get a tilt map. We demonstrate the extension using the following two examples.

**Fig. 6.**
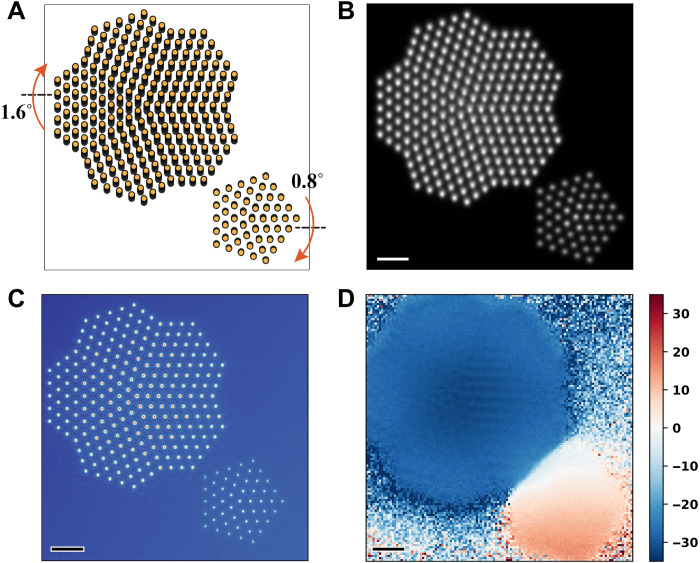
Extended APP to image copper nanoparticles with different mistilts. (**A**) Structure model of copper nanoparticles. (**B**) STEM-HAADF image. (**C**) Phase image averaged over all the four slices recovered via APP. (**D**) Recovered tilt map in the *y* direction in mrad. Scale bars, 1 nm. Poisson noise was added to the simulated CBED patterns corresponding to a dose of 1.2 × 10^5^ e/Å^2^.

First, we apply the method to copper nanoparticles. Relaxations and reconstructions of surface atoms play a key role in catalysis. Unveiling such atomic arrangement relies on high-quality atomically resolved (S)TEM images. However, because of their tiny size, nanoparticles are hard to be aligned to the electron beam direction, which hampers the atomic-scale structure analysis. The APP method offers a way to solve the problem. As shown in Fig. 6A, two copper particles were used for the 4D data simulation. The thicknesses of the larger and smaller particles are 6 and 2 nm, respectively. They are tilted 1.6° and 0.8° to two opposite directions. The sample was cut into four slices during ptychographic reconstruction. In the HAADF image (Fig. 6B), the atomic columns of the larger particle were stretched and blurred, impeding high-precision structure analysis. The extended APP method was used to correct the misorientations. High-quality phase images (Fig. 6C) were recovered together with a tilt map (Fig. 6D), reflecting different orientations of the two nanoparticles with respect to the electron beam.

**Fig. 7.**
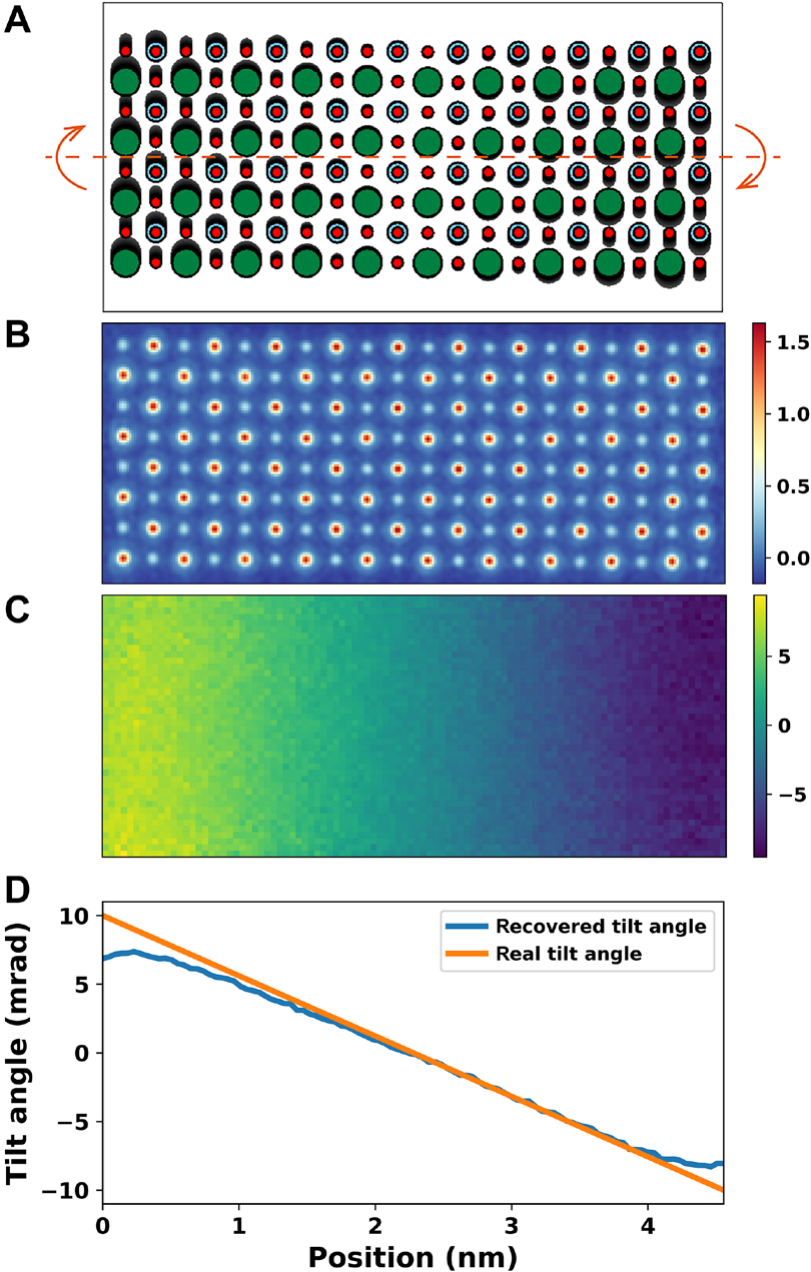
Extended APP to image structure with continuously varying tilt. (**A**) Artificial SrTiO_3_ model. The orientation continuously varies along the horizontal axis. (**B**) Phase image averaged over all the five slices recovered via APP. (**C**) Recovered tilt map in the *x* direction in mrad. (**D**) Real and the recovered tilt profiles. Poisson noise was added to the simulated CBED patterns corresponding to a dose of 1.9 × 10^4^ e/Å^2^.

Second, simulations were performed on an artificial SrTiO_3_ structure, which has continuously varying orientation (Fig. 7A). As shown in Fig. 7B, extended APP gives the zone axis projection in the whole region. The tilt map shown in Fig. 7C offers a visualization of crystal orientation distribution. The line profile of the recovered tilt map averaged along the vertical direction is in good accordance with the model. We attribute the angle deviations near the edge to the smearing effect of defocused probe, which illuminates both crystal and vacuum regions. Behaviors of extended APP under different levels of noise are shown in fig. S5. Note that the extension needs further development to improve its robustness for applications to real materials, which may include large and complex mistilt distributions in local areas.

## DISCUSSION

In summary, we have developed a reliable and efficient method to correct misorientation in electron ptychography, which is the primary experimental difficulty for deep sub-angstrom resolution imaging and quantitative atomic structure analysis of thick samples in electron microscopy. Even under large misorientations, our method can reach a spatial resolution of 0.3 Å and subpicometer accuracy in atomic structure measurement. The method makes it easier to analyze small structure distortions in crystalline materials, like some improper ferroelectrics (*49*) and high-entropy alloys (*20*). In addition, for nanoparticles (*50*) and beam-sensitive materials like zeolites (*51*, *52*), it is usually hard to accurately align the crystals to the direction of electron beam. Under these circumstances, mistilt can be relatively large and our method offers an effective way to get high-quality atomic-resolved images. Last, the extended APP shows potential in imaging materials with intrinsic orientation deviations, like those close to heterostructure interfaces, domain walls, and dislocations, as well as mapping the orientations.

## MATERIALS AND METHODS

### Experimental details

The scanning diffraction datasets were acquired using a an electron microscope pixel array detector (EMPAD) equipped on probe aberration–corrected FEI Titan Cubed Themis G2 operated at 300 kV. The convergence semiangle was set to 25 mrad. Each diffraction pattern has a dimension of 128 × 128, and the nominal camera length is 285 mm, giving a reciprocal pixel size of 0.055 Å^−1^ (1.1 mrad). Regular scanning grid was used with 64 × 64 scanning positions. About 20 nm of underfocus combined with 0.367 Å of scan step size gives an overlap ratio of about 96%. The beam current is 25 pA, and the dwell time is 1 ms. Figure S6 shows the scan geometry and the diffraction pattern. The collection angle range of the HAADF detector was 48 to 200 mrad.

### Sample preparation

The SrTiO_3_ single crystal was purchased from Hefei Kejing Materials Technology Co. Ltd. The STEM sample was prepared using the standard procedure, including mechanical grinding, dimpling, and ion milling. First, a 500-μm-thick single-crystalline sample was mechanically grinded to about 30 μm using diamond lapping films with different grit sizes. Then, the sample was stuck to a copper ring and dimpled to about 20 μm on the Gatan Dimple Grinder. Diamond slurry was used for dimpling, and aluminum oxide suspension was used for polishing. Last, the dimpled sample was milled by Ar^+^ ion on the Gatan PIPS. The accelerating voltage was set to 5 kV, and the glancing angles were ±5°. After Newton’s rings appeared, 3, 2, and 1 kV were used successively until a hole formed. The glancing angles were decreased to ±4°. Last, 0.5 kV was used to reduce the thickness of the amorphous layer.

### Multislice simulation of 4D datasets and STEM images

μSTEM (*53*) is used to simulate 4D datasets for ptychographic reconstruction. All the data are generated for the high tension of 300 kV and the convergence semiangle of 22 mrad. Other parameters are listed in table S1.

μSTEM (*53*) is also used to simulate STEM images. The thickness of SrTiO_3_ is the same as that used for simulating 4D datasets. The range of collection angle for simulated HAADF and ABF images is 48 to 200 mrad and 8 to 57 mrad, respectively. Fifty frozen lattice configurations are considered for each beam position.

### Ptychographic reconstruction parameters

The APP method is implemented using Python 3 and PyTorch (*54*). A movie is given to show the process of ptychographic reconstruction using our code on the simulated 4D dataset (movie S1). For the results shown in Fig. 3, the diffraction patterns were padded to 256 × 256 to get a real-space pixel size of 0.071 Å. Ten object slices were used for both FPP and APP reconstructions. For the results shown in Fig. 5, each diffraction pattern contains 128 × 128 pixels to get a real-space pixel size of 0.142 Å. Both drift correction and mixed-state algorithm with six probe modes were used. The phases and amplitudes of each object slice are shown in fig. S7. The probe modes for each case are shown in fig. S8.
