# Supplementary material for: Deep sub-angstrom resolution imaging by electron ptychography with misorientation correction
Source: Sci Adv. 2022 May 13;8(19):eabn2275. doi: 10.1126/sciadv.abn2275 (PMC9106290; doi:10.1126/sciadv.abn2275)
Supplement: Supplementary file 1 — Supplementary Text Figs. S1 to S10 Table S1 [file sciadv.abn2275_sm.pdf]

Science Advances
AAAS

Supplementary Materials for

Deep sub-angstrom resolution imaging by electron ptychography with misorientation correction

Haozhi Sha, Jizhe Cui, Rong Yu*

*Corresponding author. Email: ryu@tsinghua.edu.cn

Published 13 May 2022, *Sci. Adv.* **8**, eabn2275 (2022)
DOI: 10.1126/sciadv.abn2275

The PDF file includes:

Supplementary Text
Figs. S1 to S10
Table S1
Legend for movie S1

Other Supplementary Material for this manuscript includes the following:

Movie S1

Supplementary Text

Details of adaptive propagator ptychography

Among all the iterative algorithms, ptychographical iterative engine (PIE) (*24, 50*) and generalized maximum-likelihood (ML) method (*26*) are most commonly used in electron ptychographic reconstruction. Both algorithms can be extended to do multislice reconstruction. Treating the phase retrieval process as an optimization problem, one can get target parameters by various well-developed optimization algorithms such as gradient descent (GD) and Adam (*51*) method. Previous work has utilized GD to obtain the sample thickness in X-ray ptychography (*52*).

As mentioned in the article, the loss function for electron ptychography can be formulated as (*25, 26*):

$$\mathcal{L} = \sum_j \left\| |\mathcal{F}\{\varphi_{ext,j}\}| - \sqrt{I_j} \right\|_F^2 , \qquad \text{(S1)}$$

where j is the index of scanning positions. $|\cdot|$ calculates element-wise modulus of a matrix, $\mathcal{F}\{\cdot\}$ denotes the Fourier transform and $\|\cdot\|_F$ gives the Frobenius norm. φ_{ext} is the exitwave to be optimized and I is the experimentally measured diffraction intensities. When the object contains multiple slices, assuming a same propagator for all slices, the exitwave is expressed as:

$$\varphi_{ext} = \mathcal{P}_{\Delta z,\boldsymbol{\theta}}\{\dots \mathcal{P}_{\Delta z,\boldsymbol{\theta}}\{\mathcal{P}_{\Delta z,\boldsymbol{\theta}}\{P(\boldsymbol{r}-\boldsymbol{r}_j)O_1(\boldsymbol{r})\}O_2(\boldsymbol{r})\}O_3(\boldsymbol{r}) \dots\}O_N(\boldsymbol{r}) , \qquad \text{(S2)}$$

where $P(\boldsymbol{r}-\boldsymbol{r}_j)$ is the probe function shifted to the jth position and $O_{\boldsymbol{r}}^i$ is the transmission function of the ith slice. $\mathcal{P}_{\Delta z,\boldsymbol{\theta}}\{\cdot\}$ denotes the operator of Fresnel near-field diffraction which can be express as:

$$\mathcal{P}_{\Delta z,\boldsymbol{\theta}}\{\cdot\} = \mathcal{F}^{-1}\{\mathcal{F}\{\cdot\}(\boldsymbol{k})p(\boldsymbol{k};\Delta z,\boldsymbol{\theta})\} \qquad \text{(S3)}$$

$$p(\boldsymbol{k};\Delta z,\boldsymbol{\theta}) = \exp\left[-i\pi\Delta z\left(\lambda|\boldsymbol{k}|^2 - 2k_x tan\theta_x - 2k_y tan\theta_y\right)\right] , \qquad \text{(S4)}$$

where Δz is the slice thickness and (θ_x, θ_y) denotes the tilt angles between the zone axis of crystal and the incident wave. The parameters Δz and (θ_x, θ_y) are optimized by first-order gradient-based algorithms. For simplicity of calculating the gradients, only the propagator corresponding to the middle slice is considered as variable and the optimized Δz and (θ_x, θ_y) are then set to the other slices. The gradients of $\mathcal{L}$ with respect to Δz and (θ_x, θ_y) are as follows:

$$\frac{\partial \mathcal{L}}{\partial \Delta z} = 2\sum_j \sum_{\boldsymbol{q}} \mathcal{R}\{\left(\psi_j - \psi_j^{FP}\right)^* \mathcal{F}\{\varphi_{ext,j}^{\Delta z}\}\} \qquad \text{(S5)}$$

$$\frac{\partial \mathcal{L}}{\partial \theta_x} = 2\sum_j \sum_{\boldsymbol{q}} \mathcal{R}\{(\psi_j - \psi_j^{FP})^* \mathcal{F}\{\varphi_{ext,j}^{\theta_x}\}\}, \tag{S6}$$

where the sum over $\boldsymbol{q}$ denotes summing all the elements of the right matrix and $\mathcal{R}\{\cdot\}$ gives the element-wise real part of a complex matrix. (*) denotes the complex conjugate. ψ_j is the Fourier transformation of φ_{ext} and ψ_j^{FP} is the Fourier projection of ψ_j.

$$\psi_j = \mathcal{F}\{\varphi_{ext}\} \tag{S7}$$

$$\psi_j^{FP} = \frac{\sqrt{I_j}}{|\psi_j|}\psi_j \tag{S8}$$

$\varphi_{ext,j}^{\Delta z}$ and $\varphi_{ext,j}^{\theta_x}$ can be obtained in a similar way of φ_{ext}, except replacing the propagator of the middle slice with its derivates to Δz and θ_x .

$$\varphi_{ext,j}^{\Delta z} = \mathcal{P}_{\Delta z,\boldsymbol{\theta}}\{\ldots \mathcal{P}_{\Delta z,\boldsymbol{\theta}}^{\Delta z}\{\ldots \mathcal{P}_{\Delta z,\boldsymbol{\theta}}\{P(\boldsymbol{r}-\boldsymbol{r_j})O_1(\boldsymbol{r})\}O_2(\boldsymbol{r}) \ldots\}O_{N/2}(\boldsymbol{r}) \ldots\}O_N(\boldsymbol{r}) \tag{S9}$$

$$\varphi_{ext,j}^{\theta_x} = \mathcal{P}_{\Delta z,\boldsymbol{\theta}}\{\ldots \mathcal{P}_{\Delta z,\boldsymbol{\theta}}^{\theta_x}\{\ldots \mathcal{P}_{\Delta z,\boldsymbol{\theta}}\{P(\boldsymbol{r}-\boldsymbol{r_j})O_1(\boldsymbol{r})\}O_2(\boldsymbol{r}) \ldots\}O_{N/2}(\boldsymbol{r}) \ldots\}O_N(\boldsymbol{r}) \tag{S10}$$

$$\mathcal{P}_{\Delta z,\boldsymbol{\theta}}^{\Delta z}\{\cdot\} = \mathcal{F}^{-1}\left\{\mathcal{F}\{\cdot\}(\boldsymbol{k})\frac{\partial p(\boldsymbol{k};\Delta z,\boldsymbol{\theta})}{\partial \Delta z}\right\} \tag{S11}$$

$$\mathcal{P}_{\Delta z,\boldsymbol{\theta}}^{\theta_x}\{\cdot\} = \mathcal{F}^{-1}\left\{\mathcal{F}\{\cdot\}(\boldsymbol{k})\frac{\partial p(\boldsymbol{k};\Delta z,\boldsymbol{\theta})}{\partial \theta_x}\right\}. \tag{S12}$$

The equation (S5) has been first derived in X-ray ptychography (*52*) to get the thickness of samples.

Robustness of APP and FPP with respect to detector noise and overlap ratio

The robustness of APP and FPP is tested with respect to detector noise and overlap ratio. Detector noise is assumed to obey the Poisson distribution. Dwell time is assumed to be 1 ms and the beam currents are listed in fig. S1. For example, for a beam current of 0.5 pA, the CBEDs are normalized so that the total intensity of PACBED equals $0.5\times10^{-12}\times1\times10^{-3}/1.6\times10^{-19}$. Examples of PACBED patterns and single CBED patterns are shown in fig. S1. Overlap ratios listed in fig. S2 are calculated with the formula $\sigma = 1 - s/d$ where s is the scan step size and d is the probe diameter. For a defocused probe, the diameter is estimated as the product of the convergence angle and the defocus value. Ten slices are used and the slice thickness is about 2 nm. To compare the efficiency of APP and FPP, both algorithms are run 180 iterations.

Behavior of standard multislice ptychography with post alignment (FPP) under different slice thickness and regulation factor

Using the 4D datasets simulated with the multislice method, we examined the ability of FPP to correct misorientations and compared it with APP. $BaTiO_3$ of 16 nm

thick is used to generate three datasets with mistilt of 4, 8 and 12 mrad in the x direction, respectively. For each dataset, 3 different slice thicknesses (2, 1 and 0.5 nm) are tested. Post alignment of object slices is performed via cross-correlation. Multislice electron ptychography needs a regulation term to suppress the ambiguity between slices. After each iteration, the following weighting factor is applied to the Fourier transformation of 3-dimensional transmission function (*32, 53*):

$$W(\boldsymbol{k}) = 1 - \frac{2}{\pi}\arctan\left(\frac{\beta^2 k_z^2}{k_x^2 + k_y^2}\right)$$

A large regulation factor β means a wide range of low-frequency information is shared between slices. For APP, β is set to 1 and FPP is tested for β = 0.3, 0.5, 0.7 and 1.

The results of FPP heavily depend on the slice thickness and regulation factor. As shown in fig. S4A, small regulation factor gives more accurate mistilt angles. Also, to remove the misorientation effect, very thin slices (0.5 nm) are needed for FPP. However, for experimental data affected by incoherence and noise, both small regulation factor and thin slices are easy to make the algorithm fail to converge. For slices of 1 nm thick, even β = 0.3 generates about 1 mrad residual error which is large enough to stretch the atomic columns (upper parts in fig. S4C and D). As shown in the lower parts of fig. S4C and D, APP can get the mistilt angles with error smaller than 0.1 mrad for all the tested slice thicknesses. Also, as shown in fig. S4B and D, when mistilt increases, maximum phase values at the atomic sites obtained from FPP decrease even after post alignment, while for APP, maximum phase values almost remain constant.

In summary, FPP lacks the ability to correct misorientation efficiently and robustly. For a sample of 20 nm thick with a misorientation of 4 mrad, 40 slices are needed to correct the misorientation. Even the algorithm can converge, the computation time for a single run of reconstruction will be very long, let alone the thickness determination for FPP needs many times of reconstructions.

Behavior of APP to distinguish probe aberrations with mistilt

We also tested the ability of APP to get correct mistilt angles when probe aberrations like A1 and B2 exist. $BaTiO_3$ is tilted 8 mrad in the x direction away from the <001> zone axis. Poisson noise was added to the 4D dataset corresponding to 10^5 electrons per CBED. As shown in fig. S9, even with sizable A1 and B2, APP can obtain correct values of mistilt angle and sample thickness, no matter the direction of B2 is perpendicular or parallel to the mistilt direction. The optimization process corresponding to fig. S9A is shown in Movie S1.

Robustness of thickness determination using APP

For experimental dataset of Case 1, we used three different initial thickness values and the results all converged to about 23 nm (fig. S10A). Furthermore, we tested the behavior of thickness determination on the simulated data using $BaTiO_3$ of 16 nm thick. As the initial slice thickness and the number of slices vary in a range, the APP can

always obtain the correct total thickness (fig. S10B). The largest difference in total thickness between the initial value and the real value is about 24 nm, beyond which the algorithm fails to converge.

Fig. S1.

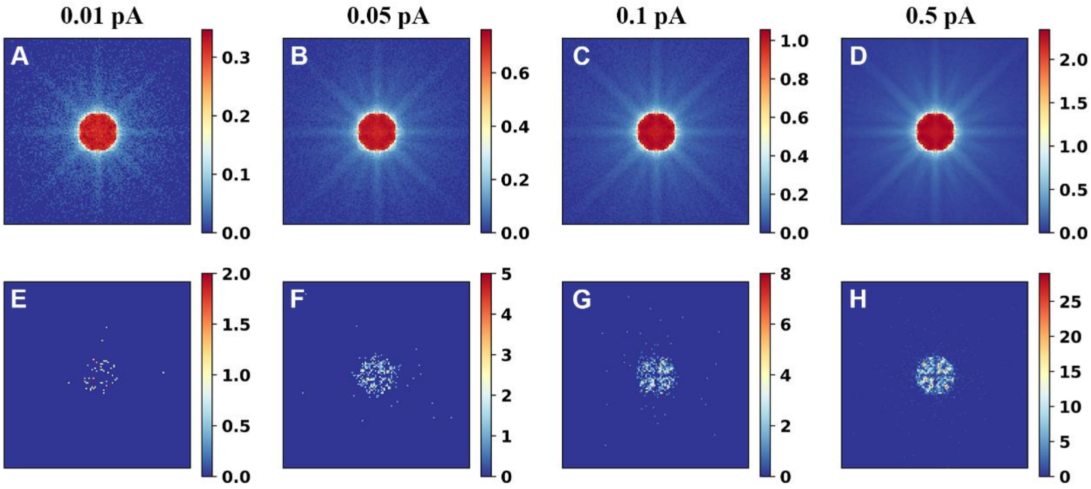

Fig. S1. (A-D) PACBED patterns and (E-H) single CBED patterns.

Fig. S2.

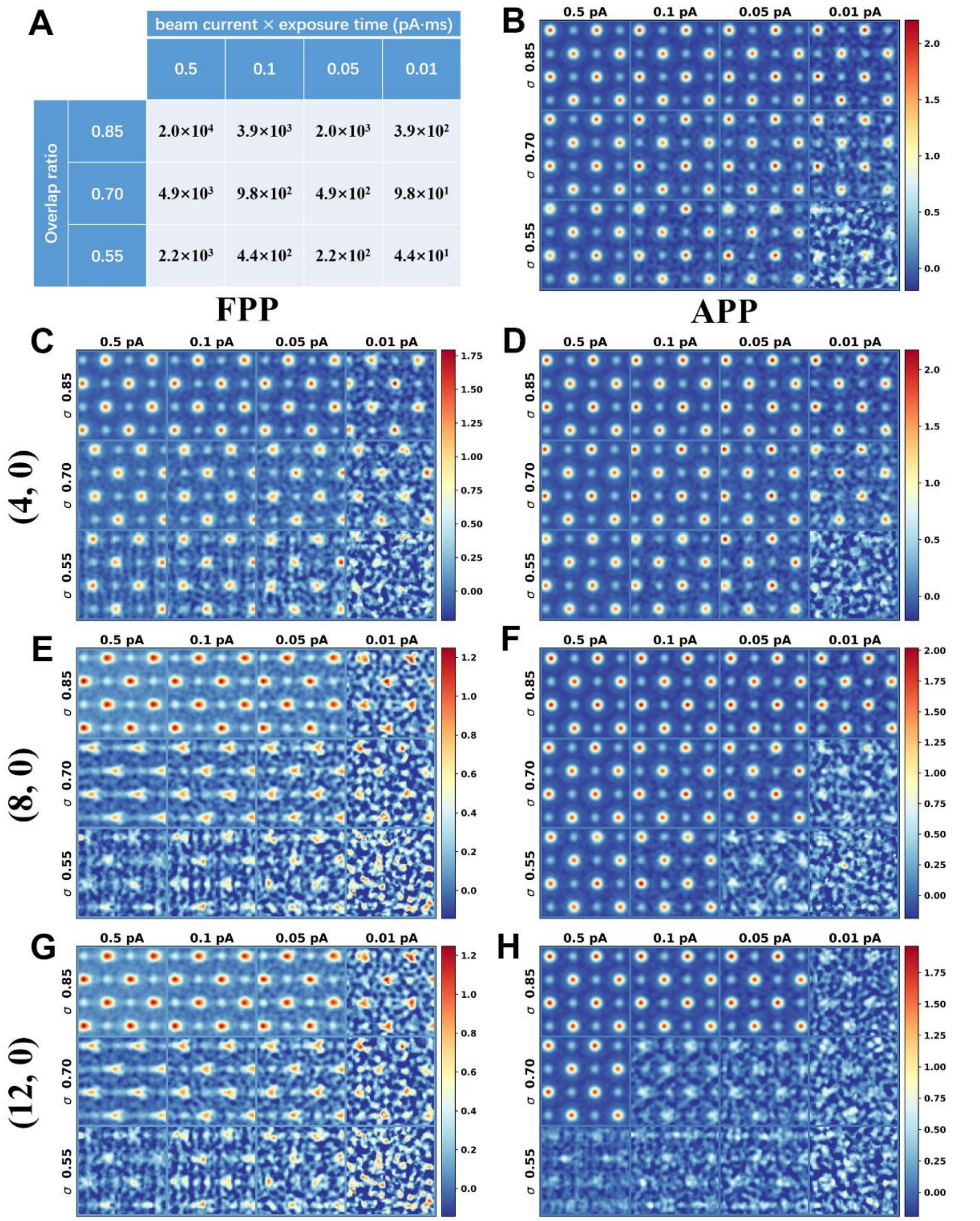

		beam current × exposure time (pA·ms)			
		0.5	0.1	0.05	0.01
Overlap ratio	0.85	2.0×10^4	3.9×10^3	2.0×10^3	3.9×10^2
	0.70	4.9×10^3	9.8×10^2	4.9×10^2	9.8×10^1
	0.55	2.2×10^3	4.4×10^2	2.2×10^2	4.4×10^1

Fig. S2. Robustness of FPP and APP with respect to detector noise and overlap ratio σ. (**A**) Dose of different conditions in this figure in e/Å^2. (**B**) No mistilt. (**C** and **D**) mistilt of 4 mrad. (**E** and **F**) mistilt of 8 mrad. (**G** and **H**) mistilt of 12 mrad.

Fig. S3.

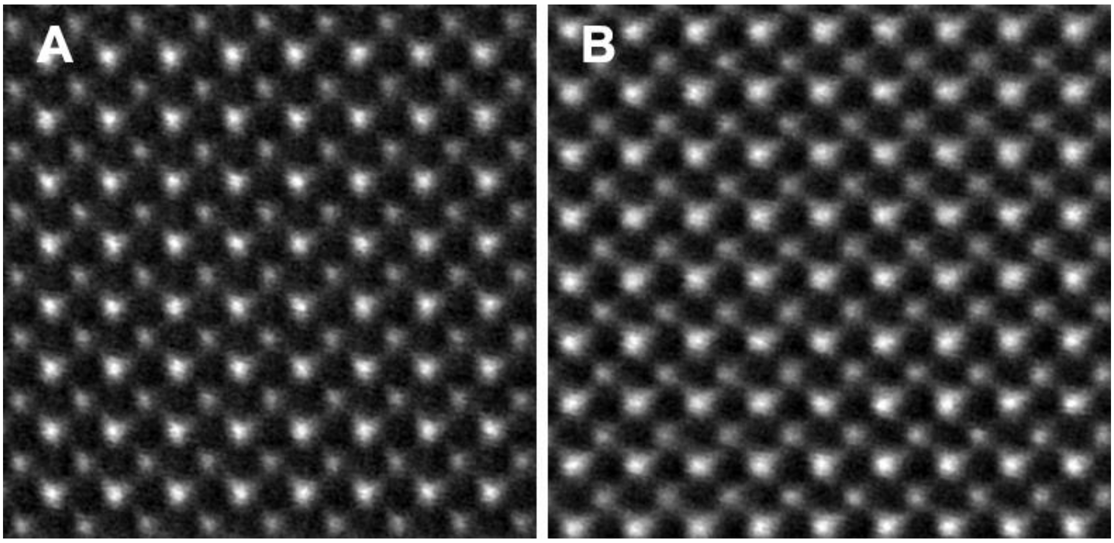

Fig. S3. HADDF images taken under the same beam current and mistilt angles for (A) Case 1 and (B) Case 2.

Fig. S4.

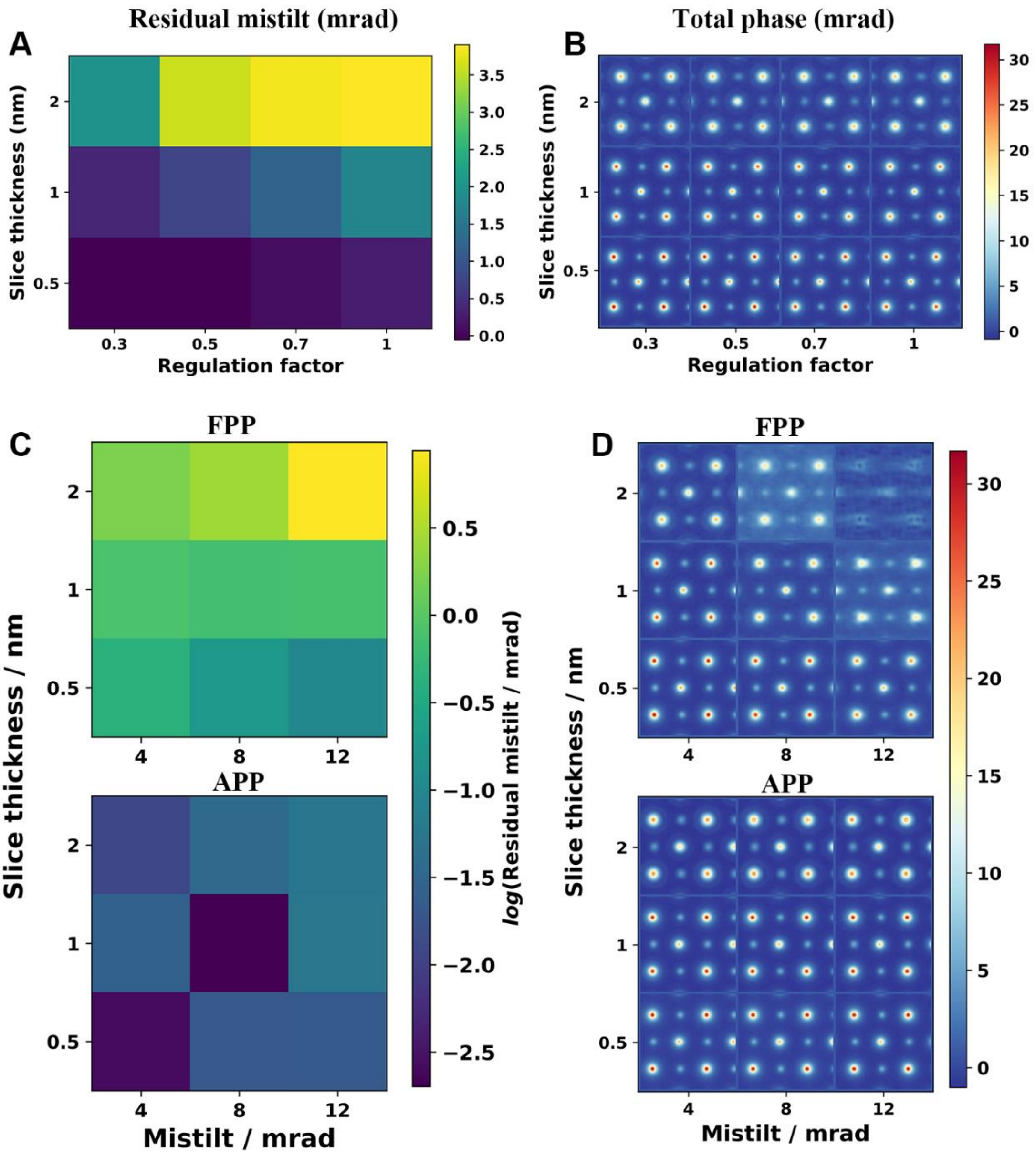

Fig. S4. Comparison of FPP and APP. (**A**) Residual errors of the mistilt angles obtained from FPP by cross-correlation at different number of slices and regulation factors. (**B**) Total phases corresponding to (A). (**C**) Residual errors of the mistilt angles obtained from FPP and APP at different number of slices and mistilt angles. (**D**) Total phases corresponding to (C).

Fig. S5.

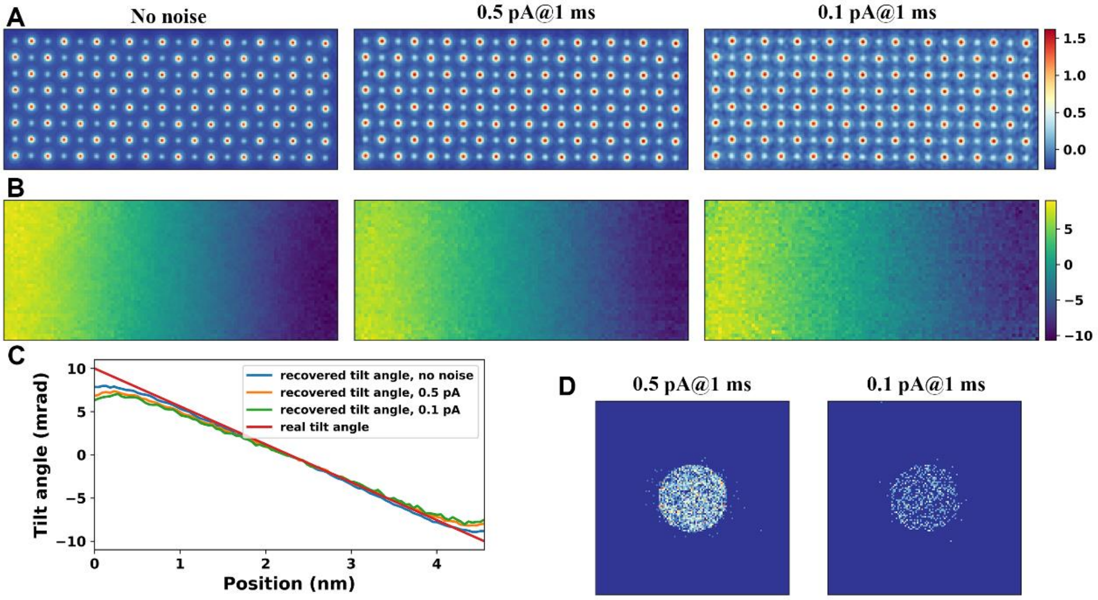

Fig. S5. Behaviors of extended APP under different levels of Poisson noise. Noise is added in the same way as results in fig. S1. (**A**) Recovered phase images averaged over all the 5 slices. (**B**) Tilt maps. (**C**) Tilt profiles averaged along the vertical direction. (**D**) Single CBEDs corresponding to beam current 0.5 pA and 0.1 pA with dwell time 1 ms.

Fig. S6.

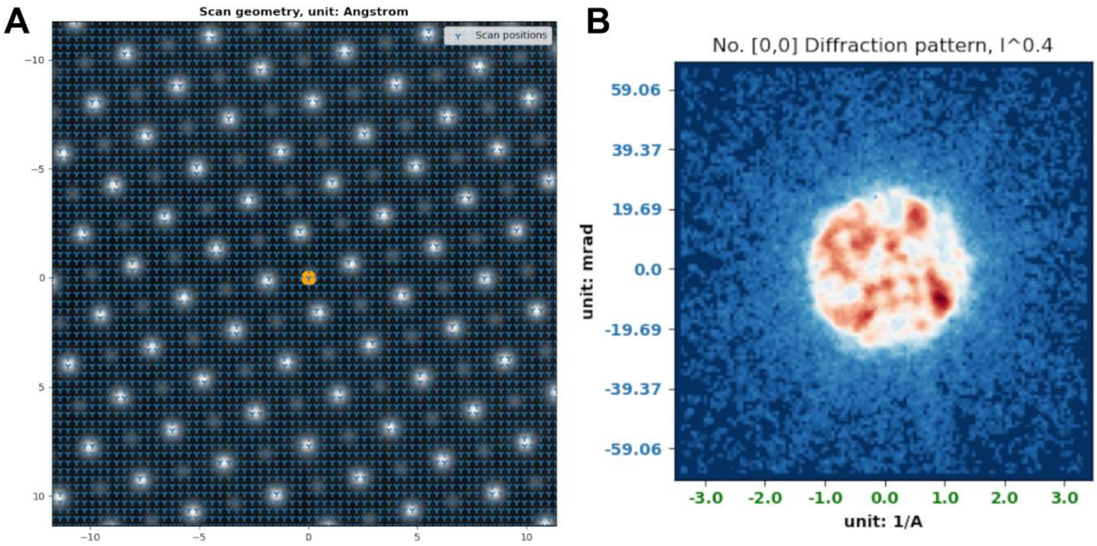

Fig. S6. Demonstration of experimental configuration. (**A**) Scan geometry of experimental datasets. (**B**) Diffraction pattern of the first scan position.

Fig. S7.

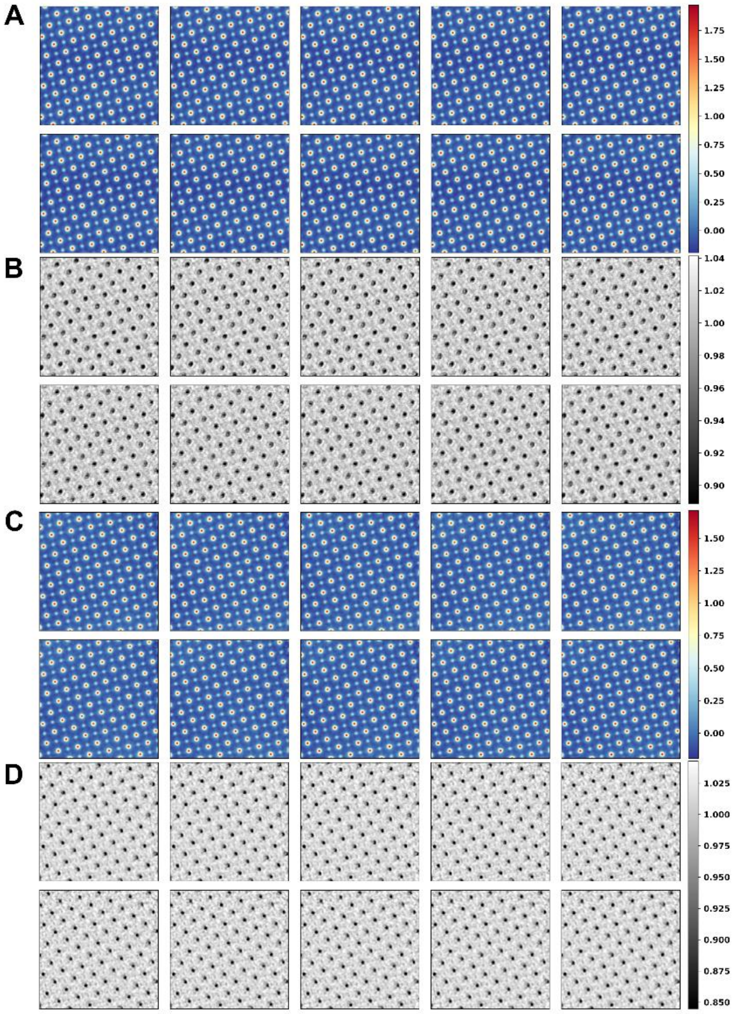

Fig. S7. Transmission functions recovered using APP. Reconstructed phases of slices for (**A**) Case 1 and (**C**) Case 2. Reconstructed amplitudes of slices for (**B**) Case 1 and (**D**) Case 2.

Fig. S8.

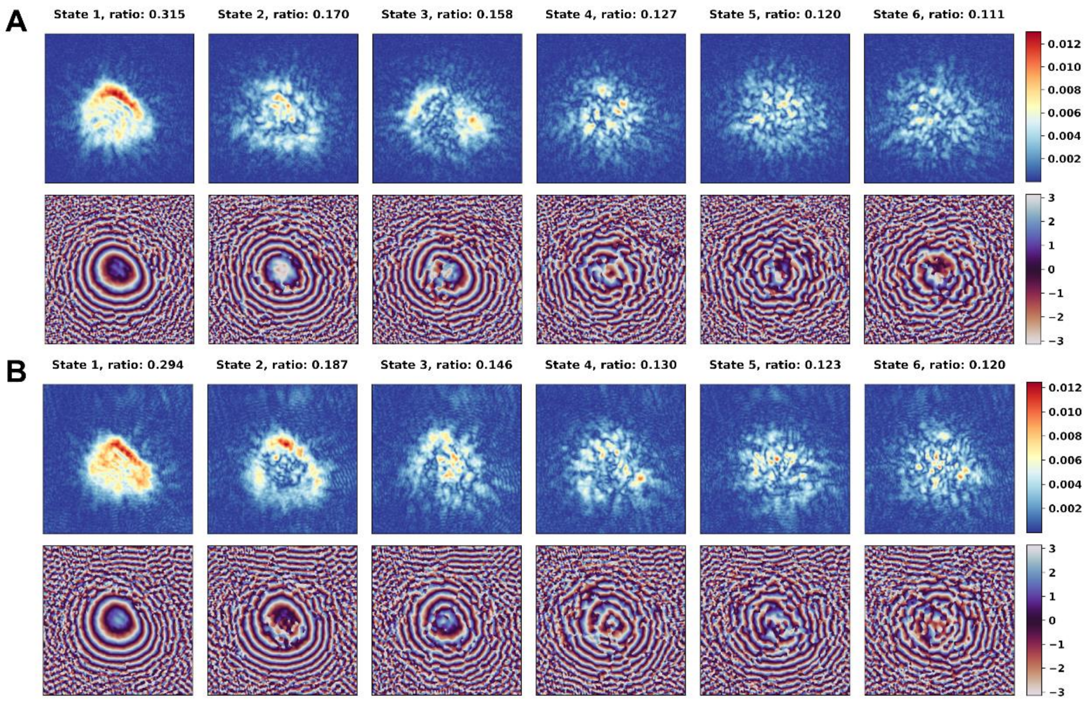

Fig. S8. Multiple states of probe functions. Reconstructed probe modes for (**A**) Case 1 and (**B**) Case 2. Ratios of each state are labeled on the top of their amplitude.

Fig. S9.

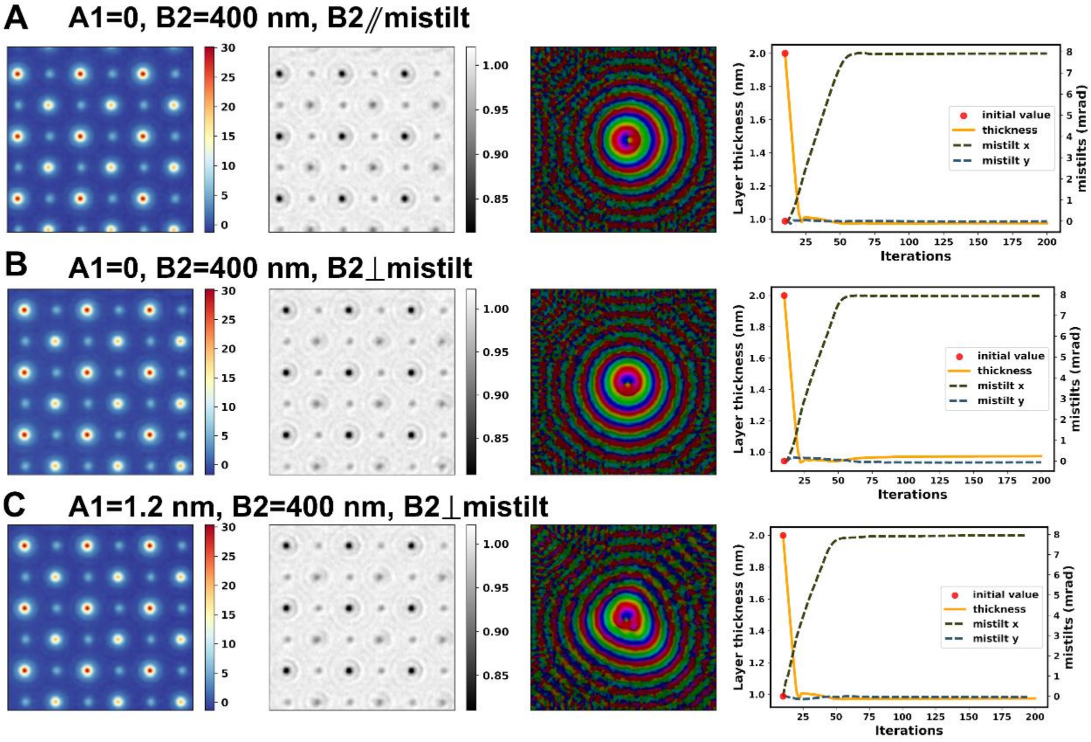

Fig. S9. APP performance in existence of astigmatism and coma. Total phases summing over all the 10 slices (the first column), average amplitudes (the second column), complex probe functions (the third column) and optimization processes of mistilt angles and thicknesses (the fourth column).

Fig. S10.

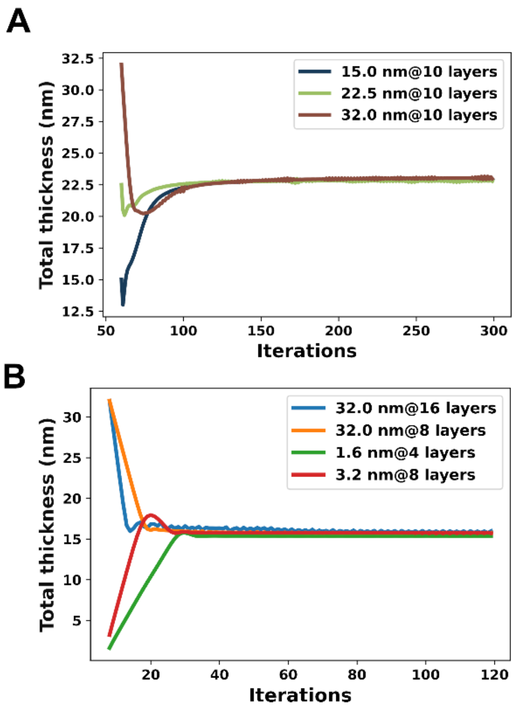

Fig. S10. Convergence of object thickness for (A) experimental dataset and (B) simulated dataset. Labels are in the form of 'total thickness @ number of slices'.

Table S1.

Structure	α	t (nm)	df (nm)	dk ($Å^{-1}$)	dim.	s (Å)	nfl	Figure(s)
$SrTiO_3$	22	20	6	0.0854	128	0.39	200	1, S1, S2
$SrTiO_3$	22	8	10	0.05	128	0.46	0	7
$BaTiO_3$	22	16	8	0.083	128	0.54	200	S4, S9, S10(B)
Cu	25	6, 2	20	0.05	128	0.50	0	6

Table S1. Simulation parameters of 4D datasets. For all 4D dataset simulations, the high voltage is 300 kV. t: thickness. df: defocus (positive value for overfocus). dk: pixel size of diffraction patterns. dim: dimensions of diffraction patterns during reconstruction. s: scan step size. nfl: the number of frozen lattice configurations repeated per scan position.

Movie S1.

Movie S1. The APP optimization process of the results shown in Fig. S9(A).
